# Evaluation of an immunomagnetic separation method to capture *Candida *yeasts cells in blood

**DOI:** 10.1186/1471-2180-8-157

**Published:** 2008-09-22

**Authors:** Véronique Apaire-Marchais, Marie Kempf, Corinne Lefrançois, Agnès Marot, Patricia Licznar, Jane Cottin, Daniel Poulain, Raymond Robert

**Affiliations:** 1 Groupe d'Etude des Interactions Hôte-Parasite, UPRES EA 3142, UFR des Sciences Pharmaceutiques et d'Ingénierie de la Santé, 16 Bd Daviers, 49045 Angers Cedex, France; 2 CHU, Laboratoire de Parasitologie-Mycologie, 4 rue Larray, 49045 Angers Cedex, France; 3 Inserm U799, Physiopathologie des Candidoses, Faculté de Médecine, Pôle Recherche, 59037 Lille Cedex, France

## Abstract

**Background:**

*Candida *species have become the fourth most-frequent cause of nosocomial bloodstream infections in immunocompromised patients. Therefore, rapid identification of pathogenic fungi to species level has been considered critical for treatment. Conventional diagnostic procedures such as blood culture or biochemical tests are lacking both sensitivity and species specificity, so development of rapid diagnostic is essential.

**Results:**

An immunomagnetic method involving anti-*Candida *monoclonal antibodies was developed to capture and concentrate in human blood four different species of *Candida *cells responsible for invasive yeast infections. In comparison with an automated blood culture, processing time of immunomagnetic separation is shorter, saving at least 24 hours to obtain colonies before identification.

**Conclusion:**

Thus, this easy to use method provides a promising basis for concentrating all *Candida *species in blood to improve sensitivity before identification.

## Background

*Candida spp. *is now the fourth most frequent cause of nosocomial blood-stream infections in critically ill patients and C*andida. albicans *is the most prevalent species [1,2]. Prevalence of other species such as C*andida tropicalis*, *Candida glabrata*, *Candida parapsilosis *and *Candida krusei *varies according to clinical conditions [3]. Rapid isolation and identification of pathogenic yeasts to species level is critical for treatment. Conventional diagnostic procedures, such as blood culture and biochemical tests lack the degree of sensitivity and specificity that would ensure reliable and early diagnosis of invasive *Candida *infections [4,5]. As a consequence, methods based on the amplification and detection of yeast DNA have been developed. Conventional PCR techniques and now real-time PCR assays are performed for specific and rapid detection and identification of fungi from clinical isolates [6]. However, as yeast load is often lower than 10 CFU per ml of blood, even in patients suffering from invasive yeast infection [7], sensitivity is the major drawback with these techniques. Sensitivity can be improved by coupling PCR method with other methods, such as hybridization assay [8], by using nested [5,9] or by optimising DNA extraction methods [10,11]. However availability of pure DNA without PCR inhibitors is essential. Another approach is to concentrate yeast cells before culture or DNA extraction using techniques such as the immunomagnetic separation method (IMS) where magnetic beads coated with monoclonal antibodies are used to capture yeast cells present in clinical specimens.

The purpose of this study was to evaluate the usefulness of IMS in improving culture yields. The effectiveness of the IMS was determined and the time to obtain colonies of *Candida *species was compared to that of the Bactec Mycosis-IC/F automated blood culture system. Human blood artificially contaminated with *C. albicans*, *C. tropicalis *which are the most frequently strains isolated in candidemia was used. *C. glabrata *and *C. krusei *were also included in the study because of their reduced susceptibility i.e. higher resistance to fluconazole.

## Results and discussion

### Analytical sensitivity of IMS

Percentages of viable yeast cells captured by beads were calculated in relation to counts of viable cells initially present in inoculated blood and expressed for 1 ml of blood (Table 1). It was observed that yeast cells of all strains studied could be recovered by IMS, but with variable recovery rates. 27.5% and 29.1% for the two strains of *C. albicans *(respectively 5575 and ATCC 66396), 38% for the two *C. glabrata *isolates (5511 and 5484) and about 15% for the two *C. tropicalis *isolates (5437 and 5395). It was moreover noted that immunoseparation recovery rates were more variable for *C. krusei *with 10.8% for isolate 5374 and 43.2% for isolate 5481. A possible explanation for the lower recovery rate of isolate 5374 is that the antigen recognised by Mab 6B3 might have been expressed at lower level in this strain. Nevertheless, IMS appears as an easy method allowing yeast cells immunocapture in less than one hour and colonies of the four *Candida *species tested after 24 h of culture on SDA-C. If IMS is coupled with a culture on chromogenic medium, it can allow the differentiation and presumptive identification of the pathogen within 24 hours. *C. parapsilosis *was not tested because it is not well recognised by the MAb 5B2. The introduction of beads coated with other specific MAbs (i.e. against *C. parapsilosis*) in the mix is expected to result in a technique for concentrating all major pathogenic *Candida *species in blood

**Table 1:** Analytical sensitivity of *Candida *cells capture with IMS method

**Strains**	**Time (hours) of positive blood culture** **(mean ± SD)**^a^	**CFU/ml in initial contaminated blood**	**CFU/ml after IMS** **(mean ± SD)**^a^	**Immunocaptured cells** **(% ± SD)**^ab^
*Candida albicans *5575	25.45 ± 0.59	32	8.8 ± 0.6	27.5 ± 1.9
*Candida albicans *ATCC 66396	25.95 ± 1.46	6	1.75 ± 0.3	29.1 ± 3.8
*Candida krusei *5374	22.26 ± 0.48	30	3.25 ± 0.5	10.8 ± 1.7
*Candida krusei *5481	16.20 ± 0.28	40	17.3 ± 2.5	43.2 ± 6.2
*Candida glabrata *5511	25.05 ± 0.5	20	7.6 ± 1.3	38 ± 6.4
*Candida glabrata *5484	25.38 ± 0.58	10	3.8 ± 0.6	38 ± 6.3
*Candida tropicalis *5437	19.02 ± 0.88	30	4.8 ± 2.2	16 ± 3.8
*Candida tropicalis *5395	17.51 ± 0.50	30	4.3 ± 2.2	14.3 ± 7.3

^a^calculation of mean and SD is derived from three different experiments

^b^percentage of immunocaptured cells: number of CFU per 1 ml of blood after IMS procedure/number of CFU per 1 ml of blood in initial contaminated blood

CFU: Colony Forming Unit, IMS : ImmunoMagnetic Separation, SD : Standard Deviation

### IMS method in comparison with conventional procedures

The IMS method has several advantages over conventional methods: it is easy to use, no special equipment is required, processing time of IMS is shorter.

In our study, with the automated blood culture method, microbial growth could be detected for all strains of *Candida *after an incubation of about 24 h (Table 1). However, the viable colonies needed to perform identification were only obtained after subsequent sub-culturing and this required a further 24-h incubation. Despite efforts made by many investigators, early and rapid diagnosis of systemic yeast infections remains limited. Culture detection of *Candida *species is often delayed or remains negative because of slow or absent growth of yeast isolates from clinical specimens. Blood cultures are positive for fewer than 50% of patients with invasive candidiasis [2,5]. Then, time to obtain colonies is shorter with IMS, saving at least 24 h in comparison with blood culture. Of course, further optimization should seek for best IMS recovery rates to increase its lower limit of detection.

In addition, as demonstrated by other authors, IMS could increase the efficiency of culture or the yield of nucleic acid before a DNA extraction. PCR assays that amplify a highly conserved sequence of the multicopy rRNA gene were developed using DNA extracts from blood specimens. 1 to 5 CFU/ml has been reported as a lower limit for DNA detection by PCR. It was shown that IMS combined with real-time PCR to detect *Plasmodium falciparum *in blood samples [12], or bacteria such as *Escherichia coli *0157:H7 and *Listeria monocytogenes *in food [13-15], or some viruses (Hepatitis A virus, Norovirus) from environmental samples or food [16,17] is particularly attractive because of the potential for concentrating micro-organisms. Therefore IMS, in conjunction with PCR, considered as one of the most sensitive methods to detect low levels of DNA from pathogens in clinical samples, could be a helpful tool in the diagnosis of candidemia.

## Conclusion

In our study, IMS has been used to capture yeast cells from artificially contaminated human blood using magnetic beads coated with monoclonal antibodies (MAb 5B2 and MAb 6B3) specific to surface antigens of *Candida*. When numbers of CFU ranged from 6 to 40 CFU per ml of blood, IMS allowed immunocapture for the four *Candida *species studied. We are fully aware that it is a study using artificially contaminated blood and another study is now in progress to evaluate what really happens in natural candidemia.

IMS should be useful in order to facilitate the isolation of yeasts from blood saving at least 24 h to obtain colonies before classic identification.

## Methods

### Strains and culture conditions

One ATCC reference strain and 7 *Candida spp. *isolated from clinical samples taken during invasive candidiasis were used: *C. albicans *ATCC 66396, 1 clinical strains of *C. albicans *(5575), two clinical isolates of each of *C. glabrata *(5511, 5484),*C. krusei *(5374, 5481), and *C. tropicalis *(5437, 5395).

Blastoconidia were grown for 24 h at 37°C on Sabouraud-dextrose-agar (SDA) containing chloramphenicol 1 mg/ml (SDA-C). Then 1 colony was suspended in 10 ml of sterile distilled water and the suspension was plated on SDA-C and incubated for 24 h at 37°C. Blastoconidia were removed with a rubber policeman and suspended into 4 ml of sterile distilled water. A dilution of 0.5 × 10^-6 ^of this suspension was prepared in distilled water to obtain a working yeast suspension and then the number of CFU was calculated by plating 100 μl of this fungal dilution on SDA-C.

### Immunomagnetic separation method versus blood culture

Immunomagnetic beads (Estapor^® ^Merck, France) coated with MAb 5B2 or MAb 6B3 (2 mg/ml) were obtained from SR2B (Avrillé, France) [18,19]. MAb 5B2 recognises the predominantly polysaccharidic antigen present on the cell surface of several species of *Candida *(*C. albicans*, *C. glabrata*, *C. tropicalis*) [20]. It is thought that the reactive structures recognised by MAb 5B2 consist of mannopyranosyl units bound through β(1–2) linkages. MAb 6B3 reacts specifically with a cell wall surface antigen that was found to be expressed by all *C. krusei *strains or isolates [19].

Following the recommendations of the manufacturer of the automated culture system that 3 to 10 ml of blood should be inoculated into Bactec Mycosis-IC/F culture vials (Becton Dickinson, Pont de Claix, France), both methods, IMS and blood culture, were carried out using samples of 4 ml of human blood for consistency.

200 μl samples of the working yeast suspension were added to 10 ml of human blood. For the blood used in this study, our institution has an agreement with the «Etablissement Français du Sang» for research experiments. After shaking, 4 ml of this contaminated blood were inoculated into Bactec fungal medium and microbial growth was detected by fluorescence (Bactec BD9240, Beckton Dickinson). When positive, samples were sub-cultured onto SDA-C medium to obtain colonies.

Separately, for the IMS procedure, 4 ml of the same yeast contaminated blood were lysed using 4 ml of lysis buffer consisting of 5 mM Tris (pH 7.5), 5 mM EDTA (pH 8) and 2% Triton X^® ^100 by shaking for 5 min at 22°C, in order to lyse erythrocytes and leukocytes and particularly polynuclear neutrophils which could contain intracytoplasmic yeasts. A mix of 50 μl magnetic beads coated with MAb 5B2 and 50 μl of magnetic beads coated with MAb 6B3, were then added. After incubation for 30 min at 22°C in an incubator (Mini Artic, Jouan, France) with gentle agitation on a sample mixer (Dynal, France), the tubes were put in a magnetic device (Dynal) in order to separate the beads from the supernatant which was discarded. The beads were then re-suspended in 100 μl of 5 mM Tris (pH 7.5), 5 mM EDTA (pH 8) before being plated onto SDA-C. After incubation for 24 h at 37°C, number of CFU, corresponding to viable cells which was obtained from 4 ml of blood were expressed per 1 ml of blood. This number of CFU per 1 ml after IMS was compared with the number of CFU determined from 1 ml of blood in the initial contaminated blood. The results were expressed in percentages of immunocaptured cells. All experiments were performed in triplicate.

## Competing interests

The authors declare that they have no competing interests.

## Authors' contributions

JC, VAM and RR conceived the study. RR gave helpful comments regarding the scientific content of the manuscript. VAM co-ordinated the study, supervised the experimental procedures and drafted the manuscript. MK and CL carried out the experiments. AML carried out the monoclonal antibodies production. PL participated in the co-ordination of the study and contributed to finalise the manuscript. DP contributed in the design of the study and will carry on this work with blood of clinical candidemia. All the authors read and approved the final manuscript.
